# Influence of Ile655Val polymorphism on trastuzumab-induced cardiotoxicity in early-stage HER2 positive breast cancer

**DOI:** 10.1038/s41598-021-93634-6

**Published:** 2021-07-13

**Authors:** Ljubica Vazdar, Ivo Darko Gabrić, Ivan Kruljac, Hrvoje Pintarić, Robert Šeparović, Lora Stanka Kirigin Biloš, Mirjana Pavlović, Ana Tečić Vuger, Mario Štefanović

**Affiliations:** 1 Department of Radiotherapy and Medical Oncology, Clinic for Tumors, University Hospital Center “Sestre Milosrdnice”, Zagreb, Croatia; 2 Department of Cardiovascular Diseases, Institute for Cardiomyopathies, Heart Failure and Valvular Diseases, University Hospital Center “Sestre Milosrdnice”, Zagreb, Croatia; 3 Department of Internal Medicine, University Hospital Center “Sestre Milosrdnice”, Vinogradska cesta 29, Zagreb, Croatia; 4 Cardiac Catheterization Laboratory, Department of Cardiovascular Diseases, University Hospital Center “Sestre Milosrdnice”, Zagreb, Croatia; 5 School of Dental Medicine, University of Zagreb, Zagreb, Croatia; 6 Faculty of Pharmacy and Biochemistry, Clinical Institute of Chemistry, University Hospital Center “Sestre Milosrdnice”, University of Zagreb, Zagreb, Croatia

**Keywords:** Breast cancer, Cancer therapy

## Abstract

Trastuzumab has improved the prognosis of HER2 positive breast cancer, but cardiotoxicity remains a concern. We aimed to identify risk factors for trastuzumab-induced cardiotoxicity, with an emphasis on the HER2 Ile655Val single nucleotide polymorphism. This single-center case–control study included 1056 patients with early-stage HER2 positive breast cancer that received adjuvant trastuzumab. Cardiotoxicity was defined as a decline in left ventricular ejection fraction (LVEF) > 15% in patients without previous cardiomyopathy, or > 10% in patients with baseline LVEF of < 50%. Patient characteristics and cardiac parameters were compared in 78 (7.38%) cases and 99 randomly assigned controls, and the polymorphism was genotyped using real-time polymerase chain reaction. Cardiotoxicity was independently associated with advanced age (*P* = 0.024), lower body mass index (*P* = 0.023), left breast involvement (*P* = 0.001), N3 status (*P* = 0.004), diabetes (*P* = 0.016), and a family history of coronary artery disease (*P* = 0.019). Genotype distribution was as follows: A/A (Ile/Ile) was found in 111 (62.7%) patients, A/G (Ile/Val) in 60 (33.9%) patients, and G/G (Val/Val) in 6 (3.4%) patients. The genotype was not associated with cardiotoxicity or the severity of heart failure, reversibility, and recovery time. We found no association between the HER2 Ile655Val polymorphism and trastuzumab-induced cardiotoxicity; therefore, we do not recommend routine cardiotoxicity-risk stratification using this polymorphism.

## Introduction

Human epidermal growth factor receptor 2 (HER2) is a transmembrane protein and a member of the epidermal growth factor receptor (EGFR) family. It is coded by the HER2/neu proto-oncogene, located on the long arm of chromosome 17 (17q12). HER2 is overexpressed in approximately 15–30% of breast cancers^1,2^, and is associated with early systemic metastases and decreased patient survival^3,4^.

Trastuzumab (Herceptin) is a humanized monoclonal antibody targeting HER2, that has dramatically improved the prognosis of HER2 positive breast cancer^5^. Trastuzumab has very few reported side effects, except for potential cardiotoxicity. Approximately 10% of patients treated with trastuzumab as a single agent show asymptomatic left ventricular ejection fraction (LVEF) decline, and 4% develop signs of congestive heart failure^6^. The mechanism responsible for trastuzumab-induced cardiotoxicity is unknown, and cardiac biopsies do not show the ultra-structural changes seen with anthracyclines, including vacuolization and myocyte cell death^7^.

Specific clinical biomarkers for the early detection of trastuzumab-induced cardiotoxicity have not been identified, and although risk factors have been examined by several groups, results have been inconsistent^8^. The promise of personalized medicine has prompted increasing research in chemotherapy pharmacogenomics, and there is evidence of a genetic component to explain the variable susceptibility to chemotherapy-induced cardiotoxicity^9^. Single nucleotide polymorphisms (SNPs) of the HER2/neu oncogene have been explored as potential markers of trastuzumab-induced cardiotoxicity, with the codon 655 [Ile655Val] polymorphism the most extensively examined. This polymorphism is a change from adenine to guanine which causes an amino acid change from isoleucine to valine.

Identifying SNPs associated with trastuzumab cardiotoxicity could help risk-stratify patients and minimize unnecessary avoidance of a proven life-prolonging therapy. Furthermore, identifying high-risk patients would ensure the timely introduction of cardiac medications known to decrease cardiotoxicity, and identify who would benefit from more frequent cardiac monitoring. Despite these potential benefits, studies examining the role of HER2 polymorphisms on trastuzumab cardiotoxicity are conflicted^10–13^. Therefore, the aim of this study was to identify risk factors for trastuzumab-induced cardiotoxicity in patients with early-stage HER2 positive breast cancer, with an emphasis on the HER2 Ile655Val polymorphism, and describe the characteristics of trastuzumab cardiotoxicity.

## Patients and methods

This single-center, case–control study was conducted at a tertiary care hospital in Zagreb, Croatia between January 2007 and December 2016. During this time, 1056 patients with HER2 positive breast cancer were treated with adjuvant trastuzumab. The studied population was predominately Caucasian. Cardiotoxicity was defined as a decline in left ventricular ejection fraction (LVEF) by > 15% in patients without previous cardiomyopathy, or > 10% in patients with a baseline LVEF of < 50%. Using these criteria, 78 (7.38%) cases were identified, and 99 randomly assigned patients without cardiotoxicity were used as controls. Patients with metastases were excluded, as these patients were more likely to receive complex chemotherapy regimens prior to trastuzumab that could increase cardiotoxicity, and indications for trastuzumab discontinuation in this group are less clear.

Following surgery, most patients received anthracycline chemotherapy regimens (doxorubicin, epirubicin), with or without taxanes (paclitaxel). Patients with an estimated increased cardiovascular risk received non-anthracycline regimens (CMF—cyclophosphamide + methotrexate + fluorouracil; paclitaxel + trastuzumab; TCH—docetaxel + carboplatin + trastuzumab; endocrine therapy + trastuzumab). If radiation was indicated, it was given after chemotherapy completion.

Trastuzumab was indicated in patients with HER2 positive breast cancer, identified using immunohistochemistry (IHC) or fluorescent in situ hybridization (FISH) in accordance with the European Society for Medical Oncology Guidelines^14^. HER2 status in tumor biopsies was determined using IHC (Hercept test), where positive results were marked as 3 + . When the Hercept test was marked as 2 + , FISH was used to confirm positivity (FISH amplification ratio ≥ 2). Adjuvant trastuzumab was given to all patients with confirmed HER2 positive breast cancer. It was given for one year, with 17 applications, divided in 3-week intervals, and was administered in a short intravenous infusion (163 patients, 92.09%) or subcutaneously (14 patients, 7.91%).

Medical records were reviewed for prior chemotherapy regimens and chest radiation, presence of cardiovascular disease, arterial hypertension, dyslipidemia, and diabetes, and family history of cardiovascular disease.

Prior to enrollment, patients were informed about the nature and aim of the investigation, and written informed consent was obtained. The study was approved by the Clinical Hospital Center “Sestre Milosrdnice” Ethics committee in accordance with the tenets of the Declaration of Helsinki (reference number: 380-59-10106-15-168/185).

### Assessment of cardiac function

A transthoracic echocardiogram (ECHO) was used to assess cardiac function. ECHO reports before and during trastuzumab treatment were retrieved from medical records. In accordance with current guidelines, baseline ECHOs were performed prior to trastuzumab therapy, with repeat ECHOs at 3-month intervals, and additional ECHOs if patients developed signs or symptoms of cardiotoxicity. If trastuzumab was discontinued due to cardiotoxicity, control ECHOs were performed every 3 to 5 weeks to assess reversibility and whether trastuzumab could be reinitiated. Complete reversibility was defined as a return of normal LVEF (LVEF ≥ 50%), and partial reversibility was defined as a 10% increase in LVEF in pateints with LVEF ≤ 50%. Diastolic dysfunction was assessed using pulsed Doppler and the transmitral flow velocity curve. Other methods were not used due to the technical limitations of devices at that time. Patients had a final ECHO after completing trastuzumab therapy.

### HER-2/neu genotyping

All patients had blood samples drawn for HER-2/neu genotyping using standard venipuncture. Deoxyribonucleic acid (DNA) was extracted from 6 ml of whole blood and ethylenediaminetetraacetic acid (EDTA) using a commercial isolation kit, the High Pure PCR Template Kit Roche (Roche diagnostics, Mannheim Germany) according to the manufacturer’s instructions. The sample of whole blood was incubated with proteinase K and lysis buffer for protein denaturation and cell membrane lysis. The denatured proteins and cellular degradation products were discarded, and the supernatant, which contained nucleic acid, was passed through columns from the test packet. The columns had a semi-permeable membrane, which retained nucleic acid, while the rest of the cellular content was eluted and discarded. The bound nucleic acid was washed twice using a wash buffer. In the final step, DNA bound to the column’s membrane was eluted in a sterile test tube using an elution buffer. The isolated DNA was stored at + 4 °C until sample analysis. The concentration of DNA isolated was around 50 ng/µL.

The HER-2/neu genotype was determined using real-time PCR (qPCR) and melting curve analysis. The PCR reaction was performed on a LightCycler 480 analyzer (Roche Diagnostics, Mannheim, Germany) using commercially available reagents, LightCycler DNA Master HybProbe (Roche Diagnostics, Mannheim, Germany), and a mixture of primers and probes, LightSNiP rs1136201 ERBB2 (HER2) (Tib Molbiol, Berlin, Germany). The 10 µl reaction mixture contained 2.5 µl of DNA, 5.2 µl H20, 0.5 µl of primers and fluorescently labeled probes, 1.0 µl FastStart DNA Master (dNTP, Mg2 + , Taq polymerase, buffer), and 0.8 µl MgCl2.

### Statistical analysis

Patient characteristics were assessed using descriptive statistics. Continuous variables were compared with one-way analysis of variance (ANOVA) and the Bonferroni method was used for post-hoc analyses. Categorical variables were analyzed using the Chi square test. Dependent continuous variables were analyzed with ANOVA for repeated measurements (RM ANOVA) and this method was used to adjust for confounding factors. Multivariate Cox regression analysis was used to analyze the association between all baseline variables and the development of cardiotoxicity and to analyze variables independently associated with recovery of cardiac function.

Power analysis was performed using data obtained from the meta-analysis published by Gómez Peña et al^13^. In this meta-analysis, cardiotoxicity was observed in 14/217 (6.45%) patients with the AA genotype and 29/113 (25.6%) patients with the AG genotype. These rates were used as input parameters for sample size calculation because the distribution of the AA and AG genotype had the allocation ratio 2:1. When considering type 1 error (α) to be 5% and type 2 error (β) to be 10%, the minimum sample size was 150 (75 patients with cardiotoxicity and 75 patients without cardiotoxicity). Two-sided p values < 0.05 were considered significant. The statistical analysis was done using IBM SPSS Version 23.0.

## Results

### Patient characteristics and cardiac function before, during, and after treatment

**Figure 1:**
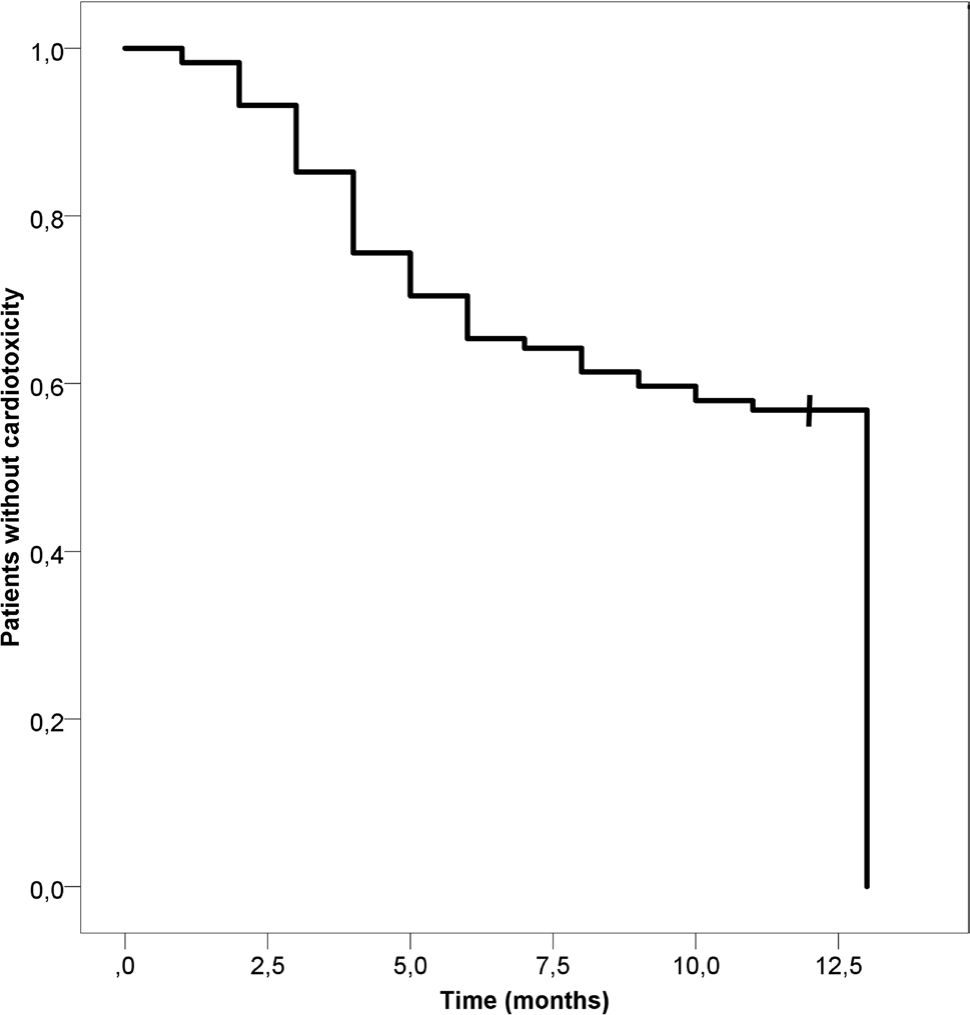
Time to develop cardiotoxicity (censored at 12 months).

The mean patient age was 59.4 ± 10.7 years and was similar in both groups. The median time to develop cardiotoxicity was 4 (3–6) months (Fig. 1). Patients that developed cardiotoxicity were more likely to have left breast involvement, N3 status, and a positive family history for heart disease (Table 1). There were no significant differences in other parameters (Table 1). In multivariate analysis, cardiotoxicity was independently associated with advanced age (HR 1.03, 95% CI 1.00–1.05, *P* = 0.024), lower BMI (HR 0.94, 95% CI 0.89–0.99, *P* = 0.023), left-sided breast involvement (HR 1.85, 95% CI 1.16–2.94, *P* = 0.001), N3 status (HR 1.44, 95% CI 1.12–1.83, *P* = 0.004), history of diabetes (HR 2.60, 95% CI 1.20–5.63, *P* = 0.016, and positive family history for coronary artery disease (HR 1.73, 95% CI 1.09–2.75, *P* = 0.019).

**Table 1:** Characteristics of patients with and without cardiotoxicity.

	Cases (n = 78)	Controls (n = 99)	*P*
Age (years)	60.9 ± 9.7	58.2 ± 11.4	0.095
BMI (kg/m^2^)	26.4 ± 4.3	27.1 ± 4.7	0.340
Hypertension n (%)	46 (59.0)	48 (48.5)	0.165
Diabetes n (%)	8 (10.3)	5 (5.1)	0.187
Dyslipidemia n (%)	23 (29.5)	20 (20.2)	0.153
Coronary artery disease n (%)	1 (1.3)	0 (0.0)	0.259
Family history of heart disease n (%)	31 (39.7)	20 (20.2)	0.004
**Genotype, n (%)**			0.934
A/A	48 (61.5)	63 (63.6)	
A/G	27 (34.6)	33 (33.3)	
G/G	3 (3.8)	3 (3.0)	
Left breast involvement n (%)	47 (60.3)	36 (36.4)	0.002
**Grade, n (%)**			0.382
1	0 (0.0)	2 (2.0)	
2	32 (41.0)	44 (44.4)	
3	53 (59.0)	53 (53.5)	
**T status, n (%)**			0.707
1	34 (43.6)	48 (48.5)	
2	37 (47.4)	46 (46.5)	
3	5 (6.4)	3 (3.0)	
4	2 (2.6)	2 (2.0)	
**N status, n (%)**			0.018
0	41 (52.6)	54 (54.5)	
1	17 (21.8)	31 (31.3)	
2	11 (14.1)	13 (13.1)	
3	9 (11.5)	1 (1.0)	
ER, n (%)	51 (65.4)	60 (60.6)	0.514
PR, n (%)	48 (61.5)	50 (50.5)	0.425
HER2 (ISH positive), n (%)	11 (14.1)	15 (15.2)	0.845
HER2 (IHC positive), n (%)	67 (85.9)	84 (84.8)	0.845
Radiotherapy, n (%)	65 (83.3)	75 (75.8)	0.218
**Chemotherapy protocol, n (%)**			0.267
4th cycle of anthracycline	22 (28.2)	22 (18.2)	
6th cycle of anthracycline	50 (62.8)	63 (62.6)	
Without anthracyclines, n (%)	6 (7.7)	12 (13.1)	0.333
**ACx4 protocol, n (%)**			0.170
ACx4 + PT	17 (81.0)	11 (61.1)	
ACx4 + Px4	5 (19.0)	7 (38.9)	

BMI, Body Mass Index; HER2, Human epidermal growth factor receptor 2; A, Adenin; G, guanine; T, Tumor; N, Node; ER, Estrogen Receptors; PR, Progesterone Receptors; ISH, In situ Hybridization; IHC, Immunohistochemistry; AC, Doxorubicin + Cyclophosphamide; PT, Paclitaxel + Trastuzumab; P, Paclitaxel, *P* value was calculated with one-way analysis of variance (ANOVA).

**Figure 2:**
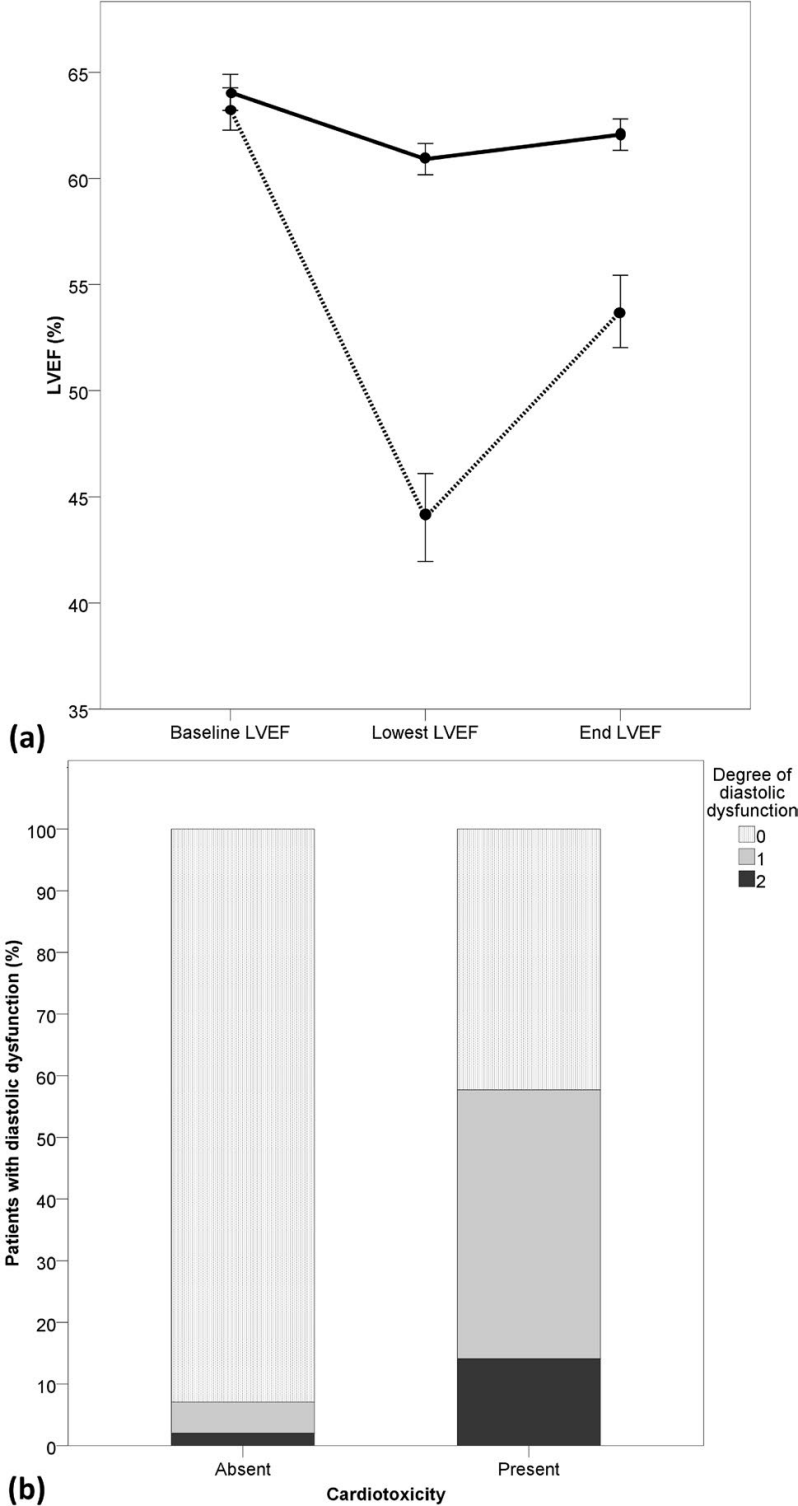
Change in systolic (**a**) and diastolic function (**b**).

Patients with and without cardiotoxicity had a mean baseline LVEF of 63.3 ± 4.4%, and 64.1 ± 4.3%, respectively. LVEF decreased significantly in both groups, but the decline in LVEF was greater in patients with cardiotoxicity (3.1 ± 4.0% vs. 19.2 ± 10%, *P* < 0.001) (Fig. 2a). Patients with cardiotoxicity had more pronounced diastolic dysfunction. First degree diastolic dysfunction was observed in 34 (43.6%) patients with cardiotoxicity and 5 (5.1%) patients without cardiotoxicity, while second degree dysfunction was present in 11 (14.1%) patients with and 2 (2.0%) patients without cardiotoxicity (Fig. 2b). Forty-eight patients (61.5%) had symptomatic congestive heart failure, the most common symptoms being palpitations (59.0%) and dyspnea (42.3%). Twelve patients (15.4%) had severe heart failure (New York Heart Association class III/IV).

**Figure 3:**
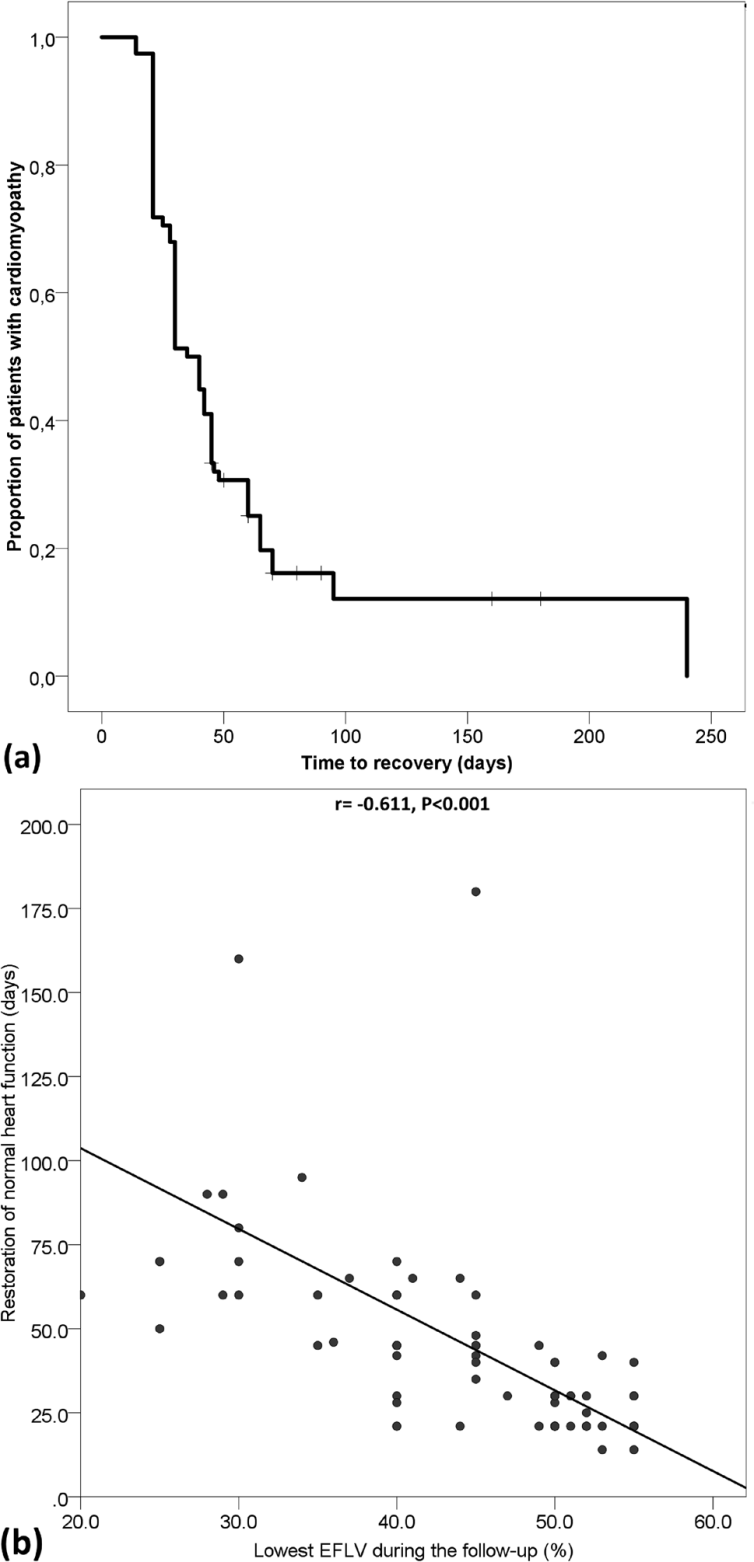
Time to recovery of cardiotoxicity (**a**) and the correlation between LVEF decline and time to recovery (**b**).

After trastuzumab treatment, LVEF increased in patients with and without cardiotoxicity, but remained lower in patients with cardiotoxicity (53.7 ± 7.6% vs. 62.1 ± 3.7, *P* < 0.001). Recovery of cardiac function was observed in 65 (83.3%) patients and the median time to recovery was 30 (21–45) days (Fig. 3a). In univariate analysis, there was a strong correlation between the lowest LVEF and time to recovery (Fig. 3b). In multivariate analysis, recovery of cardiomyopathy was independently associated with New York Heart Association (NYHA) status (HR 131.2, 95% CI 24.5–703.5, *P* < 0.001 for NYHA III/IV in predicting persistence of cardiomyopathy; HR 10.5, 95% CI 2.4–46.0, *P* = 0.003 for NYHA II), grade II diastolic dysfunction (HR 12.91, 95% CI 2.0–82.8, *P* = 0.007), and the presence of arterial hypertension (HR 1.91, 95% CI 1.05–3.33, *P* = 0.033). Recovery was not associated with age, BMI, HER2 655 variant, lowest LVEF, presence of diabetes, dyslipidemia, family history of cardiac disease, or arrhythmia.

### Association of the HER2/neu 655 polymorphism and cardiotoxicity

The HER2 codon 655 polymorphism was distributed as follows: A/A (Ile/Ile) was present in 111 (62.7%) patients, A/G (Ile/Val) in 60 (33.9%) patients, and G/G (Val/Val) in 6 (3.4%) patients. Patients with the G/G (Val/Val) variant had a statistically significant higher body mass index (BMI) (*P* = 0.049), with a non-significant trend towards a higher prevalence of arterial hypertension and positive family history of cardiovascular disease. There was no statistically significant difference in age, BMI, tumor grade, size, lymph node involvement, and hormone status in the HER-2/neu 655 variants (Table 2).

**Table 2:** Differences in cardiac function and comorbidities depending on *HER2*/*neu* genotype.

Genotype	A/A n = 111	A/G n = 60	G/G n = 6	*P*
Baseline EF, %	63.5 ± 4.2	64.1 ± 4.8	64.2 ± 2.8	0.602
Lowest EF, %	54.2 ± 10.3	52.7 ± 11.2	47.5 ± 13.7	0.323
Final EF, %	58.6 ± 7.2	58.5 ± 6.1	53.5 ± 12.5	0.368
**Diastolic dysfunction** **Baseline, n (%)**				0.228
0	66 (59.5)	33 (55.0)	1 (16.7)	
1	42 (37.8)	24 (40.0)	5 (83.3)	
2	3 (2.7)	3 (5.0)	0 (0.0)	
3	0 (0.0)	0 (0.0)	0 (0.0)	
**Diastolic dysfunction n (%)**				0.944
0	78 (70.3)	42 (70.0)	5 (83.3)	
1	25 (22.5)	13 (21.7)	1 (16.7)	
2	8 (7.2)	5 (8.3)	0 (0.0)	
3	0 (0.0)	0 (0.0)	0 (0.0)	
Hypertension, n (%)	63 (56.8)	26 (43.3)	5 (83.3)	0.078
Diabetes mellitus, n (%)	8 (7.2)	5 (8.3)	0 (0.0)	0.754
Hyperlipidemia, n (%)	27 (24.3)	13 (21.7)	3 (50.0)	0.304
Family history, n (%)	31 (27.9)	17 (28.3)	3 (50.0)	0.506
Symptomatic, n (%)	29 (60.4)	16 (59.3)	3 (100.0)	0.375
**NYHA, n (%)**				0.839
I	25 (52.1)	14 (51.9)	1 (33.3)	
II	17 (35.4)	8 (29.6)	1 (33.3)	
III/IV	6 (12.5)	5 (18.5)	1 (33.3)	
Hospitalization, n (%)	4 (8.3)	4 (14.8)	1 (33.3)	0.325
Reversibility, n (%)	42 (87.5)	21 (77.8)	2 (66.7)	0.407
Time to develop cardiotoxicity, months	4.9 ± 2.5	4.7 ± 2.8	7.0 ± 2.6	0.225
Recovery time, days	44.6 ± 33.0	48.4 ± 42.9	46.7 ± 21.4	0.817

*A* Adenin, *G* Guanine, *EF* Ejection Fraction, *NYHA* New York Heart Association, *P* value was calculated with one-way analysis of variance (ANOVA).

There was also no statistically significant difference in HER2 receptor expression and HER2/neu genotype (*P* = 0.932). A HER2 receptor expression of 3 + , measured by IHC, was present in 94 (84.7%) patients with the A/A variant, 52 (86.7%) patients with the A/G variant, and 5 (83.3%) patients with the G/G variant. HER2 receptor expression of 2 + was found in 17 (15.3%) patients with the A/A genotype, 8 (13.3%) with the A/G genotype, and 1 (16.7%) with the G/G genotype.

**Figure 4:**
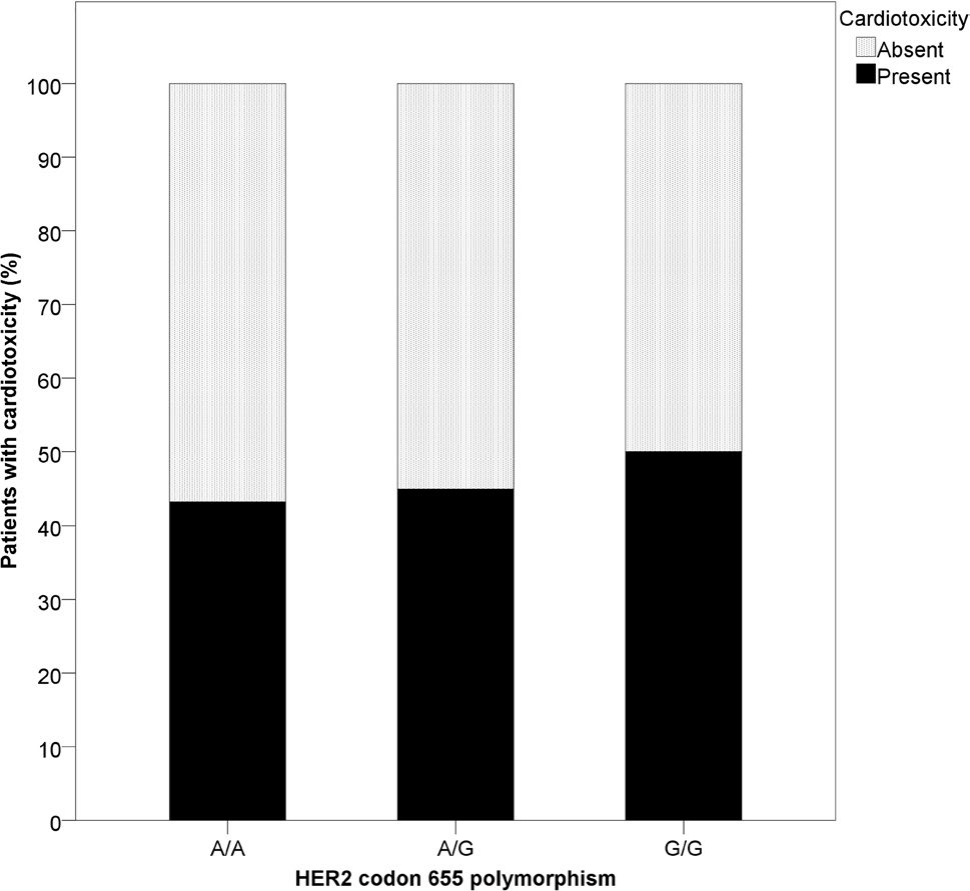
Association between HER2 655 polymorphism and cardiotoxicity.

There was no significant association between the codon 655 polymorphism and trastuzumab- induced cardiotoxicity. The A/A variant was present in 48 (61.5%) patients with cardiotoxicity, and 63 (63.6%) patients without cardiotoxicity. Variant A/G was present in 27 (34.6%) patients with cardiotoxicity and 33 (33.3%) patients without cardiotoxicity. Variant G/G was present in 3 (3.8%) patients with cardiotoxicity and 3 (3.0%) patients without cardiotoxicity. The prevalence of cardiotoxicity among each HER-2/neu genotype is shown in Fig. 4.

Patients with the G/G genotype had a somewhat greater decrease in LVEF during trastuzumab treatment, but this did not reach statistical significance (*P* = 0.323). There was no statistically significant difference in the severity of heart failure, reversibility, and recovery time in regard to the HER-2/neu genotype (Table 2).

### A subgroup analysis based on the lowest EF

Patients with cardiotoxicity were divided into two subgroups based on their lowest EF being < 50% or ≥ 50%. Like the primary analysis, there was a significant difference in terms of age, BMI, left breast involvement, N status, diabetes, and family history of coronary artery disease. Patients with lowest EF < 50% were more likely to be symptomatic and have worse NYHA functional status, have lower rates of cardiac recovery, and have longer recovery time. Trastuzumab was reinitiated in fewer patients with lowest EF < 50% and only a minority of patients completed trastuzumab treatment (Table 3). We found no differences in the HER2 polymorphism or chemotherapy protocols between the two subgroups (Table 3).

**Table 3:** Subgroup analysis of patients with cardiotoxicity based on lowest ejection fraction.

	Lowest EF < 50% (N = 43)	Lowest EF ≥ 50% (N = 35)	*P*
Age (years)	61.6 ± 8.1	60.1 ± 11.5	0.691
BMI (kg/m^2^)	26.8 ± 4.3	25.9 ± 4.3	0.329
Hypertension, n (%)	26 (60.5)	20 (57.1)	0.767
Diabetes mellitus, n (%)	4 (9.3)	4 (11.4)	0.758
Hyperlipidemia, n (%)	11 (25.6)	12 (34.3)	0.402
Family history, n (%)	20 (46.5)	11 (31.4)	0.176
**Genotype, n (%)**			0.269
A/A	25 (58.1)	23 (65.7)	
A/G	15 (34.9)	12 (34.3)	
G/G	3 (7.0)	0 (0.0)	
Left breast involvement n (%)	26 (60.5)	21 (60.0)	0.967
**Grade, n (%)**			0.767
1	17 (39.5)	15 (42.9)	
2	26 (60.5)	20 (57.1)	
3	0 (0.0)	0 (0.0)	
**T status, n (%)**			0.215
1	17 (39.5)	17 (48.6)	
2	20 (46.5)	17 (48.6)	
3	5 (11.6)	0 (0.0)	
4	1 (2.3)	1 (2.9)	
**N status, n (%)**			0.655
0	20 (46.5)	21 (60.0)	
1	11 (25.6)	6 (17.1)	
2	7 (16.3)	4 (11.4)	
3	5 (11.6)	4 (11.4)	
ER, n (%)	31 (72.1)	20 (57.1)	0.167
HER2 (ISH positive), n (%)	7 (16.3)	4 (11.4)	0.540
HER2 (IHC positive), n (%)	36 (83.7)	31 (88.6)	0.540
Radiotherapy, n (%)	35 (81.4)	30 (85.7)	0.611
**Chemotherapy protocol**			0.535
4th cycle of anthracycline, n (%)	13 (30.2)	10 (28.6)	
6th cycle of anthracycline, n (%)	28 (65.1)	21 (60.0)	
Without anthracyclines, n (%)	2 (4.7)	4 (11.4)	
**Diastolic dysfunction Baseline, n (%)**			0.845
0	22 (51.2)	20 (57.1)	
1	20 (46.5)	14 (40.0)	
2	1 (2.3)	1 (2.9)	
3	0 (0.0)	0 (0.0)	
**Diastolic dysfunction n (%)**			< 0.001
0	9 (20.9)	24 (68.6)	
1	24 (55.8)	10 (28.6)	
2	10 (23.3)	1 (2.9)	
3	0 (0.0)	0 (0.0)	
Baseline EF, %	63.3 ± 4.5	63.2 ± 4.4	0.764
Lowest EF, %	37.6 ± 7.6	51.9 ± 2.1	< 0.001
Final EF, %	51.1 ± 8.8	57.0 ± 3.6	0.001
Greatest decrement in EF, %	25.7 ± 8.5	11.3 ± 4.1	< 0.001
Time to develop cardiotoxicity, months	4.8 ± 2.9	4.9 ± 2.2	0.534
Symptomatic, n (%)	36 (83.7)	12 (34.3)	< 0.001
**NYHA, n (%)**			< 0.001
I	6 (14.0)	34 (97.1)	
II	25 (58.1)	1 (2.9)	
III/IV	12 (27.9)	0 (0.0)	
Reversibility, n (%)	30 (69.8)	35 (100.0)	< 0.001
Recovery time, days	62.3 ± 41.7	26.0 ± 7.1	< 0.001
Trastuzumab reinitiated, n (%)	15 (34.9)	33 (94.3)	< 0.001
Trastuzumab completed, n (%)	11 (25.6)	29 (82.9)	< 0.001

*BMI* Body Mass Index, *HER2* Human epidermal growth factor receptor 2, *A* Adenin, *G* Guanine, *T* Tumor, *N* Node, *ER* Estrogen Receptors, *PR* Progesterone Receptors, *ISH* In situ Hybridization, *IHC* Immunohistochemistry, *EF* Ejection Fraction, *NYHA* New York Heart Association, *P* value was calculated with one-way analysis of variance (ANOVA).

## Discussion

This study examined the characteristics of trastuzumab-induced cardiotoxicity, and the role HER2 Ile655Val polymorphism has on its development. We found no significant association between this polymorphism and cardiotoxicity (*P* = 0.934). Although there was a greater decline in LVEF in patients with the G/G genotype, it was not statistically significant (*P* = 0.323). Distribution of the polymorphism was comparable to previously published results in Caucasians^10^. Studies examining the role of this polymorphism on trastuzumab-induced cardiotoxicity are conflicted^10–13,15–17^. The first study to examine this polymorphism included 61 patients with metastatic breast cancer, and all five patients with cardiotoxicity had the Ile/Val genotype (*P* = 0.0058)^10^. Another study published as an abstract included 40 cases and 84 controls and found no difference in genotype distribution (*P* = 0.91)^15^. Lemieux et al. genotyped 73 patients with non-metastatic breast cancer and found that along with heavy alcohol intake, patients with the A/G genotype (Ile/Val carriers) were more likely to develop cardiotoxicity compared to the A/A genotype (Ile/Ile carriers) (OR = 6.00, *P* = 0.01)^11^. Similarly, a study of 78 patients also found that compared to the A/A genotype, those with the A/G genotype were more likely to develop cardiotoxicity (*P* = 0.012), and a meta-analysis combining these data with previous studies confirmed the association^13^. These differences may emerge from small sample sizes (N ranging 61–140 patients)^10,12,13,18^, cardiotoxicity definition, or ethnicity^12^. A recent, large genome-wide association study of cardiotoxicity in the N9831 trial, did not show any association with the Ile655Val polymorphism and decline in LVEF in 800 patients treated with doxorubicin, paclitaxel and trastuzumab (Arms BC)^17^. The authors explained that the lack of replication could be due to cardiotoxicity definition. Whereas a quantative model was used in the N9831 trial, previous studies used a binary definition of cardiotoxicity or no cardiotoxicity, with varying definitions. However, genotypic analyses of the AG + GG genotypes versus the AA genotype on a case control basis, using the definition of cardiotoxicity proposed by Gomez et al. (decrease in LVEF > 10% points to < 50% or any decrease > 15% or any decrease resulting in < 45% or diagnosis of CHF)^13^ showed no association (*P* < 0.05). We agree with the authors that the lack of negative studies may be a result of publication bias towards positive associations and small sample size^17^. Our study had sufficient power and a homogenous population, and we did not find this polymorphism to be a predictor of trastuzumab cardiotoxicity. However, the burden of preexisting cardiovascular comorbidities in our studied population was high, which could explain why this polymorphism did not emerge has a predictive factor.

HER2 receptor expression depends on HER2 gene amplification. This is important in tumor tissues marked as 2+ after IHC analysis of HER2 receptor expression. This is why it is mandatory to use FISH to confirm HER2 receptor positivity (FISH amplification ratio ≥ 2). Tumors labeled as HER2 2+ after immunostaining may be recategorized into HER 3+ if FISH shows an appropriate amplification ratio. Our study is the first to examine HER2 receptor intensity and HER2 genotype. We analyzed HER2 genotype separately in patients with HER 3+ after ICH analysis and those that were initially labelled as HER 2+ but were recategorized as HER 3+ after FISH. We did not find a significant association between HER2 intensity (HER2 3+ vs HER2 2+) and genotype, which suggests that HER2 intensity cannot predict genotype. This was also the first study to compare HER2 genotype and tumor grade, size, lymph node involvement, and hormone status, and we did not find any associations. Furthermore, distribution of the codon 655 polymorphism was not associated with patient age, cardiovascular risk factors (arterial hypertension, diabetes, and dyslipidemia), degree of left ventricular diastolic dysfunction, NYHA class, time to develop cardiotoxicity, or reversibility of cardiac dysfunction. A decrease in left ventricular diastolic function was observed during trastuzumab treatment (predominantly type I and II dysfunction), which has not been previously reported.

In our study, cardiotoxicity was independently associated with advanced age, lower BMI, left-sided breast involvement, N3 status, history of diabetes, and family history of coronary artery disease. Previous studies examining risk factors for trastuzumab-induced cardiotoxicity have reported conflicting results^8^, which may be due to an overall low cardiotoxicity rate, limiting sample size^8^, and different inclusion/exclusion criteria. A recent metanalysis of 17 studies showed that hypertension, diabetes, previous anthracycline use, and advanced age increased the risk of cardiotoxicity. However, because advanced age is usually accompanied by other cardiovascular comorbidities and an unfavorable lifestyle, it is unclear whether age is an independent risk factor^8^. The metanalysis did not show any significant association between trastuzumab toxicity and race, BMI, dyslipidemia, or history of coronary artery disease, and too few articles described cumulative anthracycline dosage, or left/right breast involvement to allow for statistical analysis^8^. Advanced age, diabetes, and family history of coronary artery disease are known risk factors for adverse cardiac events^19^, so it is not surprising that this was associated with cardiotoxicity in our study. We observed higher rates of left-sided breast involvement, which might be attributed to prior left chest wall radiotherapy, although studies have reported conflicting results^20,21^. Whether the association between N3 status and cardiotoxicity is due to larger radiation doses and subsequent cardiac damage is unclear.

Trastuzumab cardiotoxicity commonly manifests as an asymptomatic fall in LVEF, and in contrast to anthracyclines, does not depend on cumulative dose, is frequently reversible, and is generally well tolerated when reinitiated following cardiac recovery^22^. Previous studies have reported overt heart failure in only 1% to 4% of patients treated with trastuzumab^23–25^, with around 10% experiencing an asymptomatic fall in LVEF^24,26,27^. We observed higher rates of symptomatic heart failure (61.5%) and severe heart failure (15.4%) compared to other studies^28^. The majority of our patients received anthracycline-based protocols, which are known to increase cardiotoxicity^29^ and could explain these differences.

Cases and controls had similar baseline LVEF, and LVEF dynamics during trastuzumab treatment and were comparable to previous reports^30,31^. Similarly to previous reports^29^, cardiac recovery was observed in 83.3% of patients, with a median time to recovery of 30 days. There was a strong correlation between the lowest LVEF and time to recovery, so patients with EF below < 50% can expect longer recovery times before trastuzumab continuation. In multivariate analysis, recovery of cardiomyopathy was independently associated with NYHA status, grade II diastolic dysfunction, and the presence of arterial hypertension.

The major limitation of the study is the small sample size. Our study included a relatively homogenous Caucasian population and genetic homogeneity is needed for this type of study. Although this a strength of our study, our results may be limited to Caucasians because SNP frequencies vary by ethnicity^12^.

In conclusion, we did not find that the Ile655Val HER2 polymorphism was associated with trastuzumab-induced cardiotoxicity. Therefore, a personalized approach using this polymorphism will unlikely help risk-stratify patients. For now, the decision to use anthracycline-based regimens in patients with HER2 positive breast cancer should be carefully weighed, especially in older patients with cardiovascular risk factors, knowing that the added risk of adding such regimens to trastuzumab candidates may rob them of a proven life-prolonging therapy. Basic research is needed to better understand the mechanism of trastuzumab toxicity, which could help identify specific biomarkers of trastuzumab toxicity.

## Data Availability

The majority of data used and analyzed in the current study is included in the manuscript. The remainder is available from the corresponding author upon request.
